# A retrospective analysis of rabies post-exposure prophylaxis in Kapilvastu District, Nepal

**DOI:** 10.1186/s12889-026-27015-x

**Published:** 2026-03-14

**Authors:** Akash Adhikari, Sujan Adhikari, Abhishek Adhikari, Susma Reshmi Magar, Swagat Khanal, Sirjan Bastola, Sanjay Paudel

**Affiliations:** 1 Paklihawa Campus, Institute of Agriculture and Animal Science, Tribhuvan University, Rupandehi, Nepal; 2 Faculty of Animal Science, Veterinary Science and Fisheries, Agriculture and Forestry University, Rampur, Chitwan, Nepal; 3 Department of Veterinary Microbiology, Paklihawa Campus, Institute of Agriculture and Animal Science, Tribhuvan University, Rupandehi, Nepal; 4 Department of Basic Sciences, Paklihawa Campus, Institute of Agriculture and Animal Science, Tribhuvan University, Rupandehi, Nepal

**Keywords:** Rabies, Post-exposure prophylaxis, Animal bites, Epidemiology, Nepal

## Abstract

**Background:**

Rabies remains a fatal yet preventable zoonotic disease in Nepal, particularly in the Terai region where human-animal conflict is frequent. This study analyzes the epidemiological patterns of animal bites and evaluates the timeliness and completeness of Post-Exposure Prophylaxis (PEP) administration in Kapilvastu District to guide local public health interventions.

**Methods:**

A retrospective analysis was conducted using data from the anti-rabies vaccine register of Kapilvastu Hospital from 1 June 2022 to 31 May 2023. All recorded animal bite victims (*N* = 2,853) were included. Descriptive statistics were used to characterize demographics and exposure patterns. Multivariable logistic regression was performed to identify factors associated with delayed PEP initiation (> 24 h after exposure).

**Results:**

The study recorded 2,853 animal bite cases, predominantly males (64.5%) and children aged 0–10 years (31.4%). Dogs were responsible for the vast majority of bites (91.6%). While the PEP completion rate for the full three-dose intradermal regimen was high (93.6%), a critical delay in treatment initiation was observed; only 14.1% of victims received their first dose on the day of exposure. Multivariate analysis revealed that victims residing in municipalities outside the district headquarters had significantly higher odds of delaying treatment (AOR: 1.48; 95% CI: 1.22–1.80). Adolescents (11–20 years) were also more likely to delay PEP compared to younger children (AOR: 1.35; 95% CI: 1.07–1.70), whereas victims with high-risk bites (head/neck/hand) sought care more promptly (AOR: 0.77; 95% CI: 0.62–0.95).

**Conclusions:**

Although vaccine coverage and completion rates in Kapilvastu are commendable, the dangerous delay in initial treatment remains a critical gap in rabies prevention. Public health interventions must specifically target adolescents and remote communities to emphasize the urgency of immediate PEP, regardless of wound severity or distance to healthcare facilities.

## Background

Rabies is a fatal but preventable zoonotic disease caused by the genus lyssavirus of the Rhabdoviridae family. It affects the central nervous system of all warm-blooded animals, including mammals, and is invariably fatal once symptoms appear [1, 2]. Among the known infectious diseases, Rabies has the highest case fatality rate [3]. It is transmitted through the bite from infected animals and is prevalent in the majority of the world’s nations. Rabies can be prevented with the prompt administration of post-exposure prophylaxis (PEP) [4], adequate management of bite wounds, and public health interventions to control stray animals [5]. According to the 2018 World Health Organization guidelines, adequate PEP consists of extensive wound washing, administration of a rabies vaccine, and the infiltration of Rabies Immunoglobulin (RIG) in high-risk (Category III) cases; vaccine administration alone is considered inadequate [6]. The incubation period for rabies varies greatly, ranging from 2 weeks to 6 years [7], although it typically spans 2–3 months [8]. Once symptoms develop, Rabies is manifested with neurological signs, accompanied by excruciating pain, convulsions, violent muscular spasms, aggressiveness, hydrophobia, and photophobia [9]. A greater risk for rabies is bites on the area with rich innervation and close proximity to the brain, such as hands, neck, and face, due to a shorter pathway for the virus to move from the site of bite to reach the central nervous system (CNS) [10].

In humans, Rabies develops primarily through the bites of infected animals, and transmission of the virus occurs when the saliva of infected animals comes into direct contact with the mucus membrane and broken skin [11]. A bite from rabid animals is the predominant route of transmission of rabies, which accounts for 85–90% of human-animal bite injuries caused by dogs, and 5–10% by cats [12]. However, a slight proportion of disease in humans has been reported via wildlife such as foxes, wolves, jackals, mongooses, squirrels, and bats [13].

Animal bites represent an emerging yet neglected global public health concern. Annually, tens of millions of animal-bite injuries are reported worldwide; among all animals, mostly dogs, cats, rodents, and monkeys are the most frequently reported sources [14]. In Nepal, around 59,414 animal bite cases were reported in the year 2021/22, of which 54,996 (92.5%) were attributed to dog bite cases [15]. Stray dog bites have been identified as the primary cause of rabies in the human population [16]. Furthermore, rabies has been eradicated from the domestic dog population in the majority of industrialized countries. Other animal bites should not be neglected [17]. Globally, 59,000 human deaths are caused by rabies every year, with more than 35,000 of those occurring in Asian countries [11], over 95% of these deaths are dog-mediated and mainly predominant in countries with limited infrastructures [18]. Although safe and effective vaccines are available, complete eradication of rabies poses a significant public health challenge.

Despite ongoing national rabies control efforts, Nepal continues to face recurring rabies outbreaks. In recent years, rabies has claimed the lives of up to 32 people and 500 animals annually in Nepal, and these official figures are widely considered to be serious underestimations of the true burden due to surveillance challenges in rural areas [19]. Kapilvastu district, located in the densely populated Terai and sharing a long, open border with India, remains a high-risk area for rabies transmission. While national statistics on animal bites are available, there is a scarcity of district-level analyses that examine key programmatic indicators of PEP administration. The primary objective of this study is to analyze the epidemiological patterns of animal bites and evaluate the timeliness and completeness of PEP administration in Kapilvastu District to guide local public health interventions. These data are essential for identifying local bottlenecks and improving the effectiveness of rabies control programs to help Nepal achieve its goal of zero human deaths from dog-mediated rabies by 2030. These findings will benefit district health authorities and policymakers by identifying specific gaps in PEP delivery, enabling targeted awareness campaigns.

## Materials and methods

### Study design and period

A retrospective descriptive analysis of a clinical case series was conducted. This study design was chosen as it involves the analysis of a collection of patients with common characteristics (in this case, animal bite victims seeking PEP) to describe the epidemiological aspects of the condition over a defined period. The study utilized secondary data from all patients who presented to Kapilvastu Hospital between 1 June 2022 and 31May 2023.

### Study site and data source

The study was conducted at Kapilvastu Hospital in Taulihawa, the largest government hospital in the Kapilvastu district of Nepal. Kapilvastu is situated in the Terai plains with total population of 682,961 [20] and shares a porous open border with India to the south, which facilitates the unregulated movement of people and animals.

After receiving necessary administrative permissions, data were extracted from the hospital’s official anti-rabies vaccine register. The register systematically documents key information for each patient, including: date of bite, date of first PEP dose, patient demographics (age, sex, location), biting animal species, anatomical site of the bite, WHO category classification, and the dates of all subsequent vaccine doses administered. The classification of wound severity in the register was interpreted to correspond directly to the WHO Category I, II, and III classifications [6] used in the analysis. Data were extracted by one author using a standardized form, and a second author independently cross-checked a random sample of 10% of the records to ensure accuracy.

### Study population

The study population included all patients of any age and gender who attended the vaccination and dressing department of Kapilvastu Hospital for an animal bite during the one-year study period. A total of 2,853 patient records were included in the final analysis. As this study included all available records from the one-year study period, representing a complete census of cases presenting to the hospital, a formal sample size calculation was not required.

### Variable definitions and statistical analysis

Key variables were operationally defined for the analysis as follows:

- PEP Initiation Delay: The time from exposure to treatment was calculated as the date of the first PEP dose minus the date of the bite. Initiation “on the day of exposure” was defined as Day 0. A ‘delay’ was defined as receiving the first dose > 24 h after the bite.
- PEP Completion: Completion was defined as the documented receipt of all three doses of the anti-rabies vaccine according to the prescribed schedule.
- Adherence to Schedule: The specific three-dose intradermal regimen schedule followed at the hospital (0.1 mL at two sites on Day 0, Day 3, and Day 7) was used as the benchmark [6, 21]. A dose was considered ‘on time’ if administered on the scheduled day.

Data were entered into Microsoft Excel 2019 and analyzed using Rstudio with R (Version 4.2.0). Descriptive statistics, including frequencies, percentages, means, and ratios, were used to summarize the socio-demographic and clinical characteristics of the victims. To identify factors associated with delayed PEP initiation, a multivariable logistic regression analysis was performed. The outcome variable was binary: delayed PEP (initiation > 0 days after exposure) versus non-delayed PEP (initiation on the day of exposure). Predictor variables included age group (0–10, 11–20, 21–40, 41–60, > 60 years), sex, biting animal (dog vs. non-dog), bite site (high-risk [head/neck/hand] vs. lower-risk [leg/trunk]), WHO wound category (Category III vs. Category I/II), and geographic location (residence in Kapilvastu municipality vs. other municipalities). Both crude odds ratios (ORs) and adjusted odds ratios (AORs) with their corresponding 95% confidence intervals (CIs) were calculated. A p-value of < 0.05 was considered statistically significant.

## Results

### Socio-demographic characteristics of animal bite victims

**Fig. 1:**
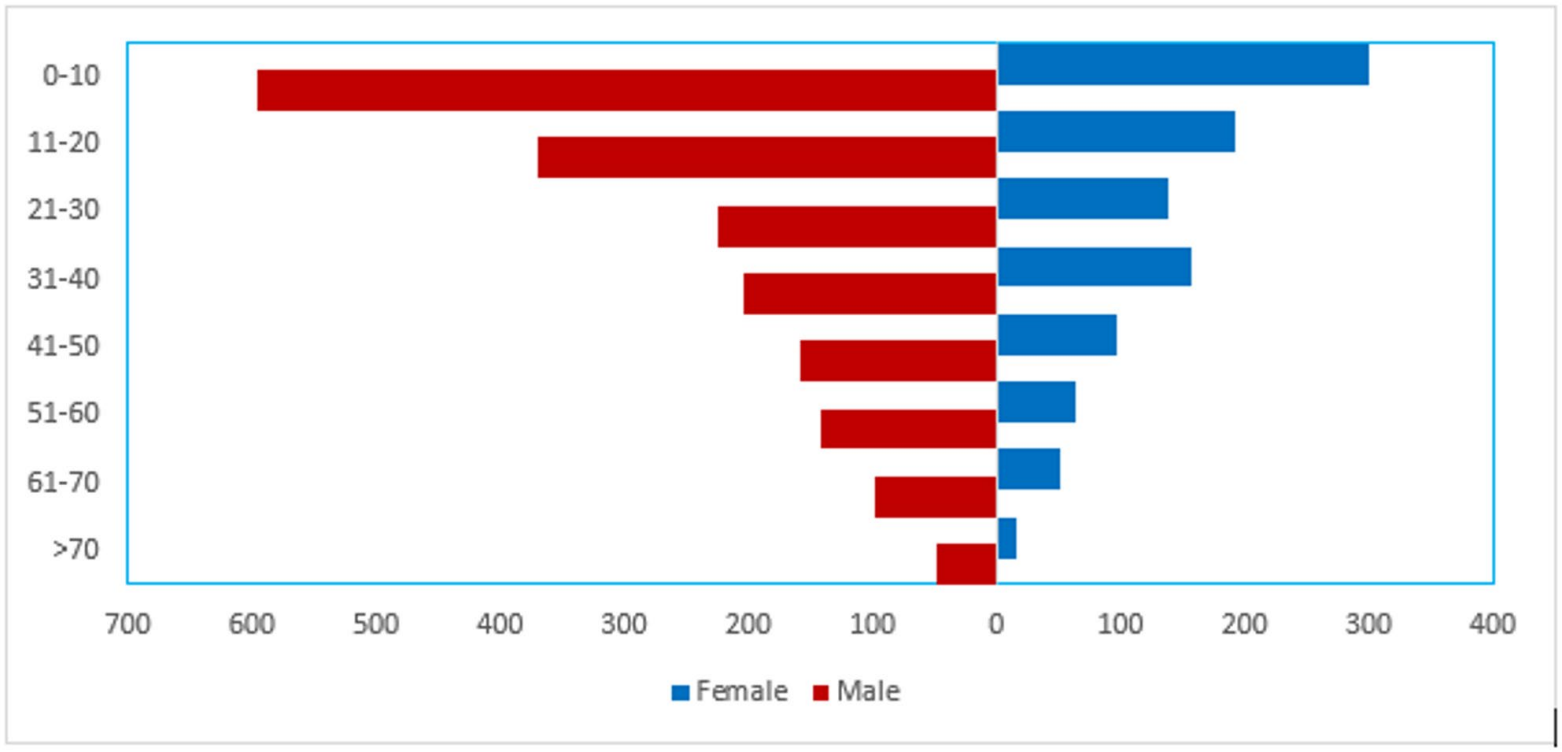
Age and sex distribution of animal bite victims

A total of 2,853 animal bite cases were recorded at Kapilvastu Hospital between June 2022 and May 2023. The incidence of bites was nearly twice as common in males (64.5%, *n* = 1,840) as in females (35.5%, *n* = 1,013), with a male-to-female ratio of 1.81:1. The burden of animal bites was disproportionately high among children. The 0–10 year age group accounted for the largest proportion of victims at 31.4% (*n* = 896). The incidence decreased with age, with individuals over 70 years being the least affected, representing only 2.2% of all cases (Fig. 1).

### Clinical and exposure characteristics

Dogs were the primary biting animal, responsible for 91.6% (*n* = 2,614) of all cases, followed by cats and monkeys. The leg was the most common site of injury, accounting for 62.7% (*n* = 1,788) of cases. The majority of exposures were classified by health workers as WHO Category II (96.0%, *n* = 2,738), with severe Category III exposures being less common (Table 1).

**Table 1:** Clinical and exposure characteristics of animal bite victims (*N* = 2,853)

Characteristic	Category	Number of Cases (*n*)	Percentage (%)
Biting Animal Species	Dog	2,614	91.6
	Cat	170	6.0
	Monkey	49	1.7
	Other	20	0.7
Site of Bite	Leg	1,788	62.7
	Hand	759	26.6
	Trunk	195	6.8
	Head/Neck	47	1.7
	Multiple Bites (> 1 bite wound)	61	2.1
	Unidentified	3	0.1
WHO Exposure Category	Category I (No exposure)	73	2.5
	Category II (Exposure)	2,738	96.0
	Category III (Severe Exposure)	42	1.5

### Geographical and seasonal distribution

**Fig. 2:**
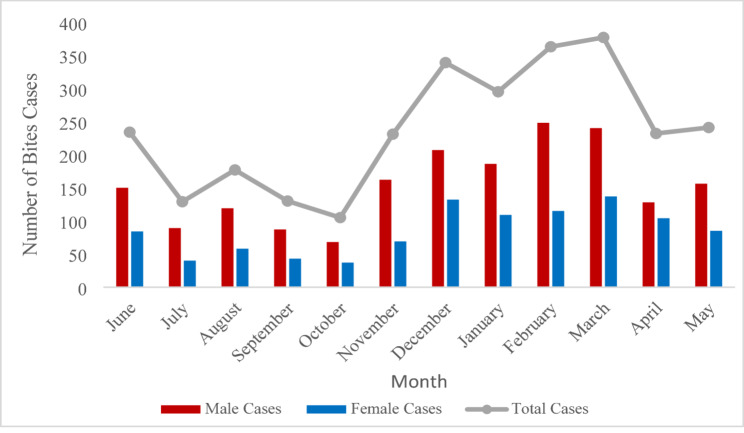
Monthly distribution of animal bite cases by sex

**Fig. 3:**
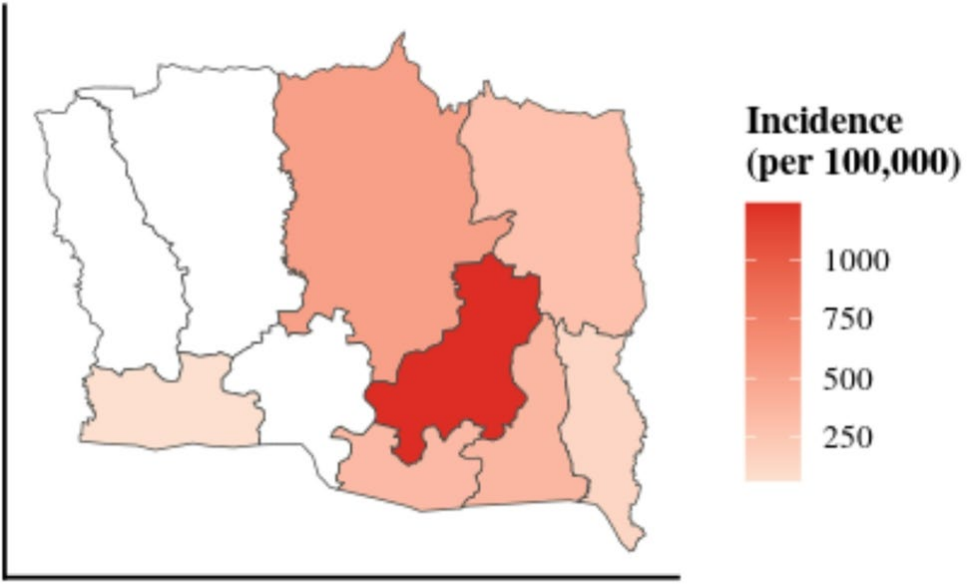
Choropleth map illustrating the spatial distribution of animal bite incidence in Kapilvastu District. The color gradient (light to dark red) represents the incidence rate per 100,000 population, with darker shades indicating a higher burden of cases

The highest number of animal bite cases occurred from January to March, suggesting a seasonal pattern (Fig. 2). A significant geographical disparity in bite incidence was also evident across the district. Kapilvastu municipality, the district headquarters, reported the highest number of cases (*n* = 1,102), corresponding to the highest incidence rate of 1,240 cases per 100,000 population. In contrast, Bijaynagar municipality reported the fewest cases (*n* = 4) and the lowest incidence rate (10 per 100,000 population) (Table 2). The spatial distribution of bite incidence across all municipalities is visualized in Fig. 3.

**Table 2:** Incidence of Animal Bites by Municipality in Kapilvastu District (June 2022–May 2023)

Municipality	Number of Cases (*n*)	Population (2021 Census)	Incidence per 100,000
Kapilvastu	1102	88,874	1240.0
Buddhabhumi	418	78,052	535.5
Maharajganj	326	65,033	501.3
Mayadevi	211	57,379	367.7
Yashodhara	181	52,693	343.5
Banganga	299	101,483	294.6
Shivaraj	200	83,124	240.6
Suddhodhan	62	45,259	137.0
Krishnanagar	50	76,727	65.2
Bijaynagar	4	40,041	10.0

### Factors associated with delayed PEP initiation

Of the 2,853 bite victims, 2,452 (86.0%) experienced a delay in initiating PEP, receiving their first dose one or more days after the exposure. The multivariable logistic regression analysis identified several factors significantly associated with this delay (Table 3).

**Table 3:** Univariable and Multivariable Logistic Regression Analysis of Factors Associated with Delayed PEP Initiation (*N* = 2,853)

Characteristic	Category	Total *N* (%)	Delayed PEP, *n* (%)	Crude OR (95% CI)	Adjusted OR (95% CI)	*p*-value
Age Group (years)	0–10	896 (31.4)	753 (84.0)	1.00 (Reference)	1.00 (Reference)	
	11–20	594 (20.8)	525 (88.4)	1.49 (1.19–1.87)	1.35 (1.07–1.70)	0.011
	21–40	776 (27.2)	672 (86.6)	1.22 (1.00-1.49)	1.14 (0.92–1.41)	0.225
	41–60	424 (14.9)	366 (86.3)	1.19 (0.93–1.52)	1.10 (0.85–1.43)	0.458
	> 60	163 (5.7)	136 (83.4)	0.96 (0.68–1.35)	0.91 (0.63–1.30)	0.596
Sex	Female	1013 (35.5)	868 (85.7)	1.00 (Reference)	1.00 (Reference)	
	Male	1840 (64.5)	1584 (86.1)	1.04 (0.86–1.25)	1.02 (0.84–1.24)	0.819
Biting Animal	Dog	2614 (91.6)	2241 (85.7)	1.00 (Reference)	1.00 (Reference)	
	Non-Dog	239 (8.4)	211 (88.3)	1.27 (0.88–1.84)	1.18 (0.80–1.74)	0.398
Site of Bite	Lower-risk (Leg/Trunk)	2034 (71.3)	1771 (87.1)	1.00 (Reference)	1.00 (Reference)	
	High-risk (Hand/Head/Neck)	819 (28.7)	681 (83.1)	0.72 (0.59–0.88)	0.77 (0.62–0.95)	0.017
WHO Category	Category I/II	2811 (98.5)	2416 (86.0)	1.00 (Reference)	1.00 (Reference)	
	Category III	42 (1.5)	36 (85.7)	0.97 (0.42–2.25)	0.91 (0.38–2.17)	0.832
Location	Kapilvastu Mun.	1102 (38.6)	912 (82.8)	1.00 (Reference)	1.00 (Reference)	
	Other Mun.	1751 (61.4)	1540 (87.9)	1.55 (1.29–1.87)	1.48 (1.22–1.80)	< 0.001

The adjusted model includes all variables shown in the table. The *p*-value corresponds to the adjusted model. For this regression analysis, patients with multiple bites (> 1 bite wound) were classified into the high-risk or lower-risk category based on their anatomically highest-risk bite site.

*OR* Odds Ratio, *CI* Confidence Interval.

Victims residing in municipalities other than the district headquarters (Kapilvastu) had 1.48 times higher odds of experiencing a delay in PEP initiation (AOR: 1.48; 95% CI: 1.22–1.80; *p* < 0.001). Age was also a significant factor; compared to the 0–10 year age group, victims in the 11–20 year group had significantly higher odds of delay (AOR: 1.35; 95% CI: 1.07–1.70; *p* = 0.011). Conversely, victims with high-risk bites (to the hand, head, or neck) had significantly lower odds of delay compared to those with lower-risk bites (AOR: 0.77; 95% CI: 0.62–0.95; *p* = 0.017). Sex and WHO wound category were not found to be significant predictors of delayed treatment initiation.

### PEP administration and adherence

The overall completion rate for the three-dose PEP regimen was 93.6%. Despite this high completion rate, a significant and dangerous delay was identified in the initiation of treatment. Only 14.0% (*n* = 401) of victims received the first dose of PEP on the same day as the animal exposure. The majority of patients (65.8%, *n* = 1,876) initiated PEP one day after being bitten, while a noTable 19.9% (*n* = 568) waited between 2 and 10 days before seeking care. The detailed distribution of delays is presented in Table 4.

**Table 4:** Distribution of time intervals between animal bite and initiation of first PEP dose (*N* = 2,853)

Time Interval	Frequency (*n*)	Percentage (%)
Day 0 (Same day of exposure)	401	14.1
Day 1 (24–48 h)	1,876	65.8
2–10 Days	568	19.9
> 10 Days	8	0.3
Total	2,853	100.0

Adherence to the subsequent vaccination schedule was stronger once patients engaged with the healthcare system. For the second dose (Day 3), 88.7% of patients were compliant with the recommended schedule, and 86.0% of patients received their third dose (Day 7) on time.

## Discussion

### Profile of animal bites and high-risk populations

The present study revealed a higher incidence of animal bites among males (64.5%) compared to females (35.5%), consistent with findings from South Bhutan where Penjor et al. reported 55.0% male victims [22] and from Iran where Gaffari-fam et al. observed 79.2% male cases [23]. This could be attributed to the differences in outdoor activities, occupational exposures, and risk-taking behavior patterns and social norms that are more common in males [17, 24]. The exposure pattern is likely influenced by cultural factors and gender roles, such that males interact with animals frequently in diverse settings. However, a study conducted among foreign tourists and expatriates in Nepal showed that the incidence was significantly higher in females than in males [24]. This suggests the variations in behavioral patterns among tourists, with females more likely to interact with animals, and underscores the importance of context when interpreting bite victim data.

Our data revealed that the children under 10 years of age were more vulnerable to animal bites, while individuals above 70 years of age were the least susceptible. The outcome is consistent with the established epidemiological patterns both within Nepal and globally. A study conducted in Nepal found that children and adolescents account for a substantial portion of the patient load [25]. A comprehensive study in India reported more than one-third of all documented dog bite victims were under 15 years old [26]. The reasons for children’s vulnerability could be due to their shorter stature, curious nature, limited information about the animal‘s behavior, and lack of experience to defend themselves effectively. The playful nature of the pediatric population with dogs and their inability to interpret their behavior could increase the risk of bites [12, 27, 28]. This emphasizes the need for educational programs for children and owners for animal safety.

The finding that dogs accounted for 91.6% of bites is a critical epidemiological insight, aligning with national data from Nepal’s Department of Health Services (92.5% dog bites) [15] and numerous international studies [29, 30]. This highlights that the primary source of rabies transmission to humans in these regions is essentially dogs, particularly free-roaming [31]. The high incidence of dog bite cases in Kapilvastu is largely due to the cultural and societal norms, such as the practice of allowing dogs to roam freely, which increases human-dog conflicts [32]. The foundation of an effective rabies prevention strategy is the comprehensive management of the dog population through three key actions: enforcing responsible pet ownership, implementing mass dog vaccinations, and managing the stray population humanely [33, 34]. While dogs are the main concern, bites from cats (6.0%) and monkeys (1.7%) are also notable rabies risks, as these animals could be potential carriers of the rabies virus.

### Wound characteristics and exposure risk

The leg (62.7%) followed by the hand (26.6%) were the predominant sites of animal bites and are consistent with epidemiological patterns reported by Pokhrel et al. [14], and Masthi et al. [35]. This distribution results from the accessibility of limbs for the bite and hands during attempts to pet, feed, or restrain an animal [36]. Children are more vulnerable to this grave concern due to their shorter stature, as bites near the head are anatomically closer to the central nervous system, potentially shortening the incubation period for rabies and increasing the risk of fatality [25, 37]. The relatively low incidence of head and neck bites highlights the importance of immediate and thorough wound care for any bite, because of the critical nature of these areas for rabies transmission [38].

The majority of bite cases in this study were classified as Category II (96.0%). This suggests that most reported animal exposures presented in Kapilvastu Hospital consist of minor scratches without bleeding or bruising of exposed skin. While Category III bites were less common (1.5%), they represent the highest risk for rabies transmission and require urgent PEP. This discrepancy with studies by Kinge & Supe [39] and Sadasivan et al. [40] which reported a majority of Category III bites, could be due to differences in population characteristics, animal behavior, or reporting biases [41].

### Temporal and geographical patterns of bite incidence

In this present study, the highest number of animal bite cases occurred from January to March, suggesting a seasonal pattern. This seasonal peak during the cooler, post-winter months supports the conclusion of previous findings in Nepal [25]. The primary explanation for this pattern is linked to the dog reproductive cycle, as the major breeding for dogs in Nepal occurs around October [42]. The two-month gestation period results in a surge of new litters born between January and March. During this period, lactating female dogs exhibit pronounced protective and aggressive behaviors, significantly increasing the likelihood of bite incidents [23, 43]. Further, these seasonal trends likely coincide with increased outdoor activities, including agricultural practices, which elevate the frequency of human-animal interactions [44]. An understanding of these seasonal patterns is crucial for resource allocation and the timing of public awareness campaigns, especially before and during peak months.

The geographical disparity in bite incidence was evident, ranging from a high of 1102 cases in Kapilvastu municipality to a low of just 4 in Bijaynagar. This could be due to several factors, including population density, stray animal populations, level of awareness, reporting mechanisms, and accessibility to healthcare facilities [45, 46]. The higher burden in Kapilvastu suggests a consistent effort is needed in this area, including mass dog vaccination campaigns, stray animal birth control programs, and public awareness initiatives from the local and central levels. In contrast, the lower incidence in Bijaynagar might warrant further investigation to ensure accurate reporting and access to services.

### Post-exposure prophylaxis: successes and critical gaps

A key positive finding was the high rate of PEP completion, with 93.6% of animal bites victims receiving the full three-dose course of the anti-rabies vaccine. This is an admirable public health achievement, especially surpassing completion rates reported in other research (65% reported by Sahu et al. [47], 83% reported by Penjor et al. [22]). This indicates increased community awareness regarding the fatal nature of rabies, robust public health campaigns, with consistent availability of vaccines in hospitals, and commitment of health care workers in providing counselling to local people [18]. This high completion rate is a cornerstone for preventing rabies deaths. To achieve the goal of zero deaths, however, efforts must now target the barriers preventing the final 6.43% of patients from completing the full course.

Despite this success, our findings indicate a significant and dangerous delay in administration of 1st dose. Only 14.1% of victims received the first dose on the same day of exposure, and 19.9% waited for 2 to 10 days, compromising the time-sensitive efficacy of the treatment. The data aligns with Gaffari-fam et al. [23], attributing similar factors: individual observing animal behavior, lack of immediate awareness, or logistical challenges such as longer travel distances to health facilities [27, 48]. The failure to administer PEP within the golden hour following exposure significantly increases the risk of clinical disease progression.

Following the initiation of PEP, 88.7% of bite victims received their 2nd dose on time, and 86.0% received their 3rd dose on time. This indicates that once patients are engaged in the healthcare system, the majority are committed to completing their treatment. This sustained adherence is a positive indicator of both patient motivation and the reliability of the service at Kapilvastu Hospital. However, there were notable proportions (11.3% and 14.0% respectively) who failed to adhere to the recommended schedule. This highlights systemic challenges such as limited access to facilities, public holidays, or lack of time availability that need to be addressed [49]. To improve adherence to complete vaccinations, it’s crucial to make the process easier and more supportive. These could include flexible clinic hours, sending appointment reminders, engaging community health workers for follow-ups, and ensuring a steady, uninterrupted vaccine supply so that no one misses a dose due to stockouts. Also, exploring the mechanism for vaccine delivery closer to communities, especially in remote areas, can make a big difference [45]. Engaging community health workers for follow-up and patient tracking, especially for those in remote areas, could also prove highly effective in closing this adherence gap and ensuring that every patient receives the full, life-saving benefit of the PEP course [41, 50]. In addition, this study was based on routinely collected medical records. It is important to stress that this study describes patients that sought care in health facilities, and therefore it missed a critical population that did not seek medical attention.

The increased odds of delay among adolescents (11–20 years) compared to younger children (0–10 years) is a notable finding. While younger children are often under closer parental supervision, adolescents may have more autonomy, potentially leading them to downplay or hide bite incidents to avoid reprimand or the inconvenience of treatment. Conversely, the fact that high-risk bites (to the hand, head, or neck) were associated with more prompt treatment suggests that the perceived severity of a wound is a key motivator for seeking immediate care [44]. This highlights a critical gap in public awareness: the community may not fully appreciate that any bite, regardless of location or apparent severity, requires urgent medical attention.

### Limitations

This study has several limitations that should be considered. First, as a single-center, hospital-based case series, the findings are subject to selection bias and may not be generalizable to all animal bite victims in the district, particularly those who sought care elsewhere or not at all. Second, the analysis relies on secondary data from a routine clinical register, which may be subject to data entry errors or inconsistencies. Additionally, because the register focuses primarily on vaccine administration, detailed clinical records concerning pre-hospital wound washing practices and the infiltration of RIG in Category III cases were unavailable for analysis. Finally, this study documents the administration of PEP, not the prevention of rabies; it cannot determine the true incidence of rabies in the biting animal population or the number of human cases successfully averted. Future prospective research is needed to explore the specific socioeconomic and logistical reasons for the observed delays in seeking care.

## Conclusion

This study confirms that animal bites remain a significant public health problem in Kapilvastu district, disproportionately affecting males and young children, with dogs constituting the majority of animal bites. While the completeness of the three-dose regimen was high, the vast majority of victims failed to receive their first dose on the day of the bite. Therefore, while the vaccination program itself is robust, future public health initiatives must urgently focus on educating the community about the critical need for immediate medical consultation after any animal bite to close this dangerous time gap and prevent rabies fatalities.

## Data Availability

The anonymized dataset generated and analyzed during the current study is available from the corresponding author upon reasonable request. To protect patient confidentiality, specific identifiers have been removed from the dataset.

## Abbreviations

AOR: Adjusted Odds Ratio
CI: Confidence Interval
CNS: Central Nervous System
PEP: Post-Exposure Prophylaxis
WHO: World Health Organization
